# NIR Spectroscopy for Discriminating and Predicting the Sensory Profile of Dry-Cured Beef “Cecina”

**DOI:** 10.3390/s20236892

**Published:** 2020-12-02

**Authors:** Isabel Revilla, Ana M. Vivar-Quintana, María Inmaculada González-Martín, Miriam Hernández-Jiménez, Iván Martínez-Martín, Pedro Hernández-Ramos

**Affiliations:** 1 Food Technology, University of Salamanca Escuela Politécnica Superior de Zamora, Avenida Requejo 33, 49022 Zamora, Spain; avivar@usal.es (A.M.V.-Q.); miriamhj@usal.es (M.H.-J.); ivanm@usal.es (I.M.-M.); 2 Analytical Chemistry, Nutrition and Bromatology, University of Salamanca Calle Plaza de los Caídos s/n, 37008 Salamanca, Spain; inmaglez@usal.es; 3 Graphic Expression in Engineering, University of Salamanca Escuela Politécnica Superior de Zamora, Avenida Requejo 33, 49022 Zamora, Spain; pedrohde@usal.es

**Keywords:** near infrared spectra, chemometry, dry meat, artificial neural networks, organoleptic parameters, prediction, protected geographical indication distinguishing

## Abstract

For Protected Geographical Indication (PGI)-labeled products, such as the dry-cured beef meat “cecina de León”, a sensory analysis is compulsory. However, this is a complex and time-consuming process. This study explores the viability of using near infrared spectroscopy (NIRS) together with artificial neural networks (ANN) for predicting sensory attributes. Spectra of 50 samples of cecina were recorded and 451 reflectance data were obtained. A feedforward multilayer perceptron ANN with 451 neurons in the input layer, a number of neurons varying between 1 and 30 in the hidden layer, and a single neuron in the output layer were optimized for each sensory parameter. The regression coefficient R squared (RSQ > 0.8 except for odor intensity) and mean squared error of prediction (MSEP) values obtained when comparing predicted and reference values showed that it is possible to predict accurately 23 out of 24 sensory parameters. Although only 3 sensory parameters showed significant differences between PGI and non-PGI samples, the optimized ANN architecture applied to NIR spectra achieved the correct classification of the 100% of the samples while the residual mean squares method (RMS-X) allowed 100% of non-PGI samples to be distinguished.

## 1. Introduction

The dry-cured beef meat called Cecina is a meat of intermediate moisture from different anatomic parts which undergoes a process of profiling, salting, washing, settling, smoking and drying; the whole procedure takes between 7 and 12 months after salting. In Spain, cecinas produced exclusively in the province of León from hind leg pieces of beef cattle (at least 5 years old and 400 kg of weight) and produced following the processing scheme established by the Supervisory Council of cecina de León may be awarded the quality label “Protected Geographical Indication” (PGI) [1]. The most remarkable characteristics of this product include its intense red color, smoked odor, slightly salty taste, and soft texture owing to which it is highly appreciated by consumers. These sensory characteristics constitute the distinguishing marks of this product. One of the activities of the Supervisory Council of PGI cecina de León is, therefore, to perform a sensory analysis to determine the existence of defects and to certify its typicality in order to differentiate it from that of non-PGI products [2].

However, a descriptive sensory analysis is a very complex and time-consuming process which involves the recruitment, selection, training and qualification of assessors following the ISO 8586 and ISO 5492 standards [3,4]. Although it is possible to find literature on the creation of sensory panels to certificate Protected Denomination of Origin (PDO) or PGI products [5,6], the information related to meat products is scarce [7]. This illustrates the difficulty of performing a sensory analysis for products under quality labels and means that it would be desirable to replace it with a fast, reliable, and cheap instrumental analysis.

Near infrared spectroscopy (NIRS) is a fast, accurate, multiparametric and non destructive technique which, due to its agility and can be implemented on-line. This technique has been shown to be a powerful tool for discriminating products according to the geographical origin of the samples. Therefore, it has been used for rice wines [8], honey [9], tea [10], and lentils [11] among other products. These results indicate that this technique can be a simple way of distinguishing PGI from non-PGI meat products. However, there are numerous discriminating methods which could be applied to NIR spectra which have been assayed for discriminating between food products belonging to PDO or PGI quality labels. Examples include principal component analysis for almonds [12], factorial discriminant analysis (FDA) for Swiss cheeses [13], partial least squares discriminant analysis (PLS-DA) for vinegars [14], K-nearest neighbors (KNN) for hazelnuts [15], the residual mean squares method (RMS-X residuals) for dry sausages [16] or artificial neural networks (ANN) for cheeses [17]. However, all this research has also shown that the most suitable distinguishing method is closely linked to the product; a detailed study should be carried out for each specific PDO or PGI label or food matrix.

Moreover, NIRS has also shown promising results for predicting the sensory characteristics of meat [18,19] and meat products such as sausages [20] or dry-cured ham [21,22,23]. However, a determinant step is the selection of the chemometric tool for multivariate analysis of data. In this sense, previous studies indicate that ANNs are more suitable for predicting sensory parameters than multiple regression tools. In general, higher regression coefficient R squared (RSQ) and lower mean squared error of prediction (MSEP) are observed when ANN are used for predictions [24,25,26] and it is possible to predict a higher number of parameters [16].

This study is part of a larger project which aims to develop a fast, objective, and reliable methodology for classifying and predicting the sensory parameters of meat products with quality labels [20,23]. Taking this into account, the aim of this study was to assess the feasibility of the prediction of sensory parameters of dry-cured beef meat cecina using NIR spectroscopy, as a fast and non destructive method, together with Levenberg–Marquardt feedforward ANN of the multi-layer perceptron type, which have shown the best results for other meat products. Moreover, the study also aims to determine the most suitable multivariate classification tool for distinguishing whether a sample belongs to a quality label such as the PGI Cecina de León only by using its NIR spectra.

## 2. Materials and Methods

### 2.1. Samples

Fifty samples of dry-cured beef Cecina were used for this study. Of these, 25 samples belonged to the “cecina de León” Protected Geographical Indication and were produced according to the specifications of the Regulatory Board of the cecina de León PGI published in the Official Bulletin of Castilla y León [27]. To this end, the hind leg was selected and after trimming the external connective tissue the pieces were covered in salt, placed in piles alternating meat and salt, and kept at 3–4 °C and 85–90% relative humidity (RH) for 3 days. The pieces were subsequently rinsed to remove the salt and moved again to the cold-storage room under controlled temperature and humidity for 50 days’ post-salting. The smoking process was carried out using oak wood in a smoking chamber (5 days at 12–15 °C and 65–75% RH). Finally, the pieces were dried and aged by reducing the RH from 80–85% to 60–65% at 12–16 °C for a variable period of time. The total period of salting, rinsing, post-salting, smoking, drying and aging ranged between 7 and 12 months.

The remaining 25 samples did not belong to the PGI and were acquired in shops in the same province (León) as those of the PGI and their productive process was similar to that of “cecina de León”.

### 2.2. Sensory Analysis

The panel that carried out the sensory analysis was formed by 10 assessors with a wide previous experience of dry-cured meat analysis as previously described [16,23]. The training for the specific sensory profiling of this product involved 8 sessions lasting 1–1.5 h each. The choice, definition and consensus of the evaluation methodology of the parameters were established during four sessions.

The selected parameters for appearance were veined, fat color, color intensity, exudate and white dots. For flavor, the evaluated parameters were odor intensity, cured odor, rancid odor, flavor intensity, cured flavor, saltiness, sweetness, rancidity and aftertaste. Finally, for texture the parameters were hardness, juiciness, fibrousness, chewiness and gumminess. The description and score criteria for these parameters were the same as previously selected for dry-cured ham by this sensory panel [23]. Together with these parameters, the following which were characteristic of dry-cured beef meat were also selected by the assessors: smoked odor and smoked flavor described as intensity of odor and flavor respectively produced by the presence of smoke, moldy odor described as the presence of a characteristic odor recalling that of mushrooms, and pungency described as the intensity of a pungent sensation. Other parameters such as color homogeneity, atypical aroma, fat flavor intensity, sourness, atypical flavor, heterogeneity of the texture, or chewing residue which were assessed in dry-cured ham were not included by the panel for dry-cured beef meat characterization.

During the next four qualification sessions, the same sample was assessed three times each session. The results were used to calculate the reproducibility of the panel whose maximum uncertainty has to be lower than 0.5 and repeatability of the panel whose maximum uncertainty has to be lower than 0.8 [5]. Finally, 14 quantification sessions were held with four samples being tested in each of them. Some selected samples were tasted three times to check the continuous accuracy of the panel. A slice of approximately 1.5 mm thick from each sample was presented to the assessors. The samples were coded with three digits codes and kept at room temperature. A structured 9-point scale was used for each of the attributes, ranged from low intensity (1) to high intensity (9) of the parameter.

A statistical analysis of the sensory data was carried out in the form of an analysis of variance (ANOVA) using the SPSS Package 25 (IBM, Chicago, IL, USA).

### 2.3. Near Infrared (NIR) Spectroscopy

**Figure 1:**
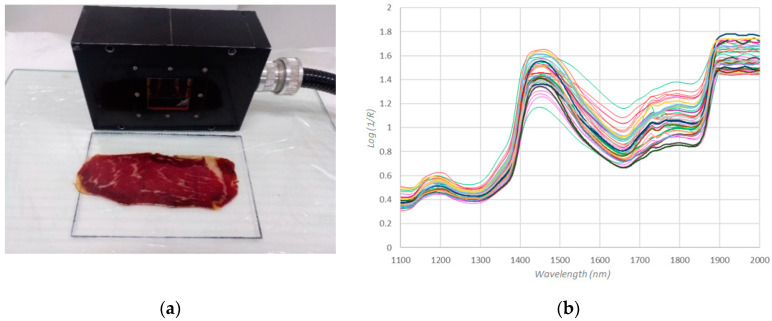
Near infrared (NIR) measurements: (**a**) a record of NIR Spectra; (**b**) spectra of the cecina samples.

All samples were analyzed by using Foss NIR System 5000 equipment (Foss, Hillerod, Denmark) to obtain the NIR spectra. The NIR measurement was recorded by applying directly the quartz window of 5 cm × 5 cm to a slice of the product cut with a slicing machine transversally to the direction of the muscle fiber (Figure 1a).

The NIR System is coupled with a remote reflectance fiber-optic probe of 1.5 m 210/210 (Ref no. R6539-A) and uses a remote reflectance system and a ceramic plate as a reference. The spectra of the samples were recorded between 1100 and 2000 nm (Figure 1b), at intervals of 2 nm, i.e., 451 data for each spectrum; 32 scans for both the reference and the samples were recorded for each sample. Three NIR records were made for each slice of cecina, one in the centre of the slice and the other two at the ends of the slice. The average representative spectrum of each slice of cecina was correlated with the sensory data provided by the sensory panel, as the assessors were given the whole slice and consumed it completely for evaluation. Spectra were stored as the logarithm reciprocal of reflectance log (1/R) (R = reflectance). The software used for spectral collection and data handling was Win ISI 1.50 (Foss, Hillerod, Denmark) installed on a Hewlett-Packard Pentium III computer.

### 2.4. Discriminant Analysis

Samples of cecina were analyzed according to whether they belonged to the “cecina de León” PGI or not. The significance of the effect of belonging to the PGI as determined by sensory attributes was assessed by the analysis of variance (ANOVA). Indeed, different discriminant analysis was carried out using the whole NIR spectrum from 1100 to 2000 nm measuring each 2 nm, i.e., 451 data.

Soft independent modelling of class analogy (SIMCA) using principal component analysis (PCA) was used to group the samples. The data for SIMCA modeling were normalized, scaled, and mean-centered. Subsequently the original variables were linearly transformed into a new set of variables (principal components, PCs) which preserve the information of the original set. The number of PCs for classifying purposes was determined by selecting those with an eigenvalue of > 1. The projection of the samples in the space determined by the principal components allowed the detection of groups present in the samples. An orthogonal projection latent structure discriminant analysis (OPLS-DA) was then carried out. To do so, the total of the samples were randomly divided into a training set (80% of the data) and a validation set (20% of the samples) in order to test the robustness of the discriminant model. Moreover, it was possible to calculate the discriminating plot which identifies the wavelengths or bands which may have a higher impact on the classification ability of the model. The software used was SIMCA-P software version 14.1 (Umetrics, Sartorius Stedim Biotech AS, Umeå, Sweden).

The RMS-X residuals analysis was carried out with the Win ISI 1.50 (Infrasoft International, State College, PA, USA) software using the whole NIR spectrum. Different combinations of the following mathematical treatments (none, multiplicative scatter correction (MSC), standard normal variate (SNV), detrend (DT) or SNV-DT), first or second derivatives, and several gaps over the derivative were calculated, and different numbers of data points in a running average and one or two smoothing were assayed and coded as follows (None 2,10,10,1) as previously described by González-Martín et al. [28]. The best mathematical treatment for distinguishing between the samples was selected taking into account the highest percentage of correctly classified samples.

The ANN selected for product classification was a multi-layer perceptron feedforward of the backpropagation type. This ANN type uses the tangent sigmoid function in the hidden layer and the softmax transfer function in the output layer. The following ANN learning or training algorithms were tested in order to minimize the error process: gradient descent, gradient descent with adaptive learning rate backpropagation, gradient descent with momentum, gradient descent with momentum and adaptive learning rate, scaled conjugate gradient, conjugate gradient with Powell–Beale restarts, conjugate gradient with Fletcher–Reeves restarts, conjugate gradient with Polak–Ribiere restarts and Levenberg–Marquardt.

For each of the learning algorithms the ANN architecture includes an input layer with 451 data, a hidden layer with a number of neurons between 1 and 30 and one output layer with two nominal variables that was PGI or non-PGI. If the classification is in the correct class output the target output value is 1 and 0 for the other nominal variable. The data (NIR spectra) set was randomly divided into three sets: the training set with 70% of the data, the validation set with 15%, and the test set with 15%. For all the ANN structures 500 trainings with different initial seed values were held in order to select the ANN with the best performance, which was established by the highest percentage of correctly classified samples. The software used was the Deep Learning Toolbox of MatLab (MathWorks^®^) in its R2020a version.

### 2.5. Artificial Neural Network for Predicting Sensory Parameters

In this case, a feedforward artificial neural network of the multi-layer perceptron type was used for processing the data. The input layer had 451 neurons (i.e., 451 values of log (1/R) recorded by NIR spectroscopy), a varying number of neurons between 1 and 30 were also tested in the hidden layer, and the output layer had only one neuron that showed the predicted value of the sensory parameter. An ANN was constructed for each of the 24 sensory parameters. As previously reported by Hernández-Jiménez et al. [16], the best training algorithm for predicting sensory parameters is the Levenberg-Marquardt backpropagation. The hyperbolic tangent sigmoid function was selected for the hidden layer and the pure linear transfer function was used for the output layer. The weight and bias matrix were randomly initialized and a known seed value number was used and stored. As previously reported, this will allow the reproducibility of the ANN data [17]. For all the ANN, the data (NIR-expected sensory parameter) were randomly divided into three sets: the training set with 70% of the data, the validation set with 15%, and the test set with 15%. In order to achieve the best prediction capability, 100 trainings of architectures were tested for each sensory parameter. The best ANN architecture was selected according to the highest value of the RSQ and the lowest value of the MSEP. The software used was the Deep Learning Toolbox of MatLab (MathWorks^®^) in its R2020a version.

## 3. Results and Discussion

### 3.1. Sensory Data

The mean values together with the minimum, maximum and standard deviation for the 50 samples analyzed are shown in Table 1. These values show that the range of variation was wide enough to guarantee an adequate margin for calibration purposes. The observed variation is a consequence of using different producers, ripening times, and pieces of muscle [2,29].

The sensory parameters were divided into three groups: appearance parameters, flavor parameters including odor, flavor and taste characteristics and texture characteristics to coincide with previous studies which used these three modalities for the attributes of cecina [2]. Regarding appearance parameters, veined showed a mean between the mean values previously reported for beef cecina [2,29]. Mean values for fat color and color intensity were nearer to those described by Rodríguez-Lázaro et al. [2], while the mean value for exudate, equivalent to brightness of lean described by these authors, was slightly lower. The amount of white spots was not previously quantified for cecina but is a parameter that is usually applied in dry-cured ham descriptions [7,22,30]. These white crystals appear during the meat curing process and are composed mainly of tyrosine [31]. The mean value was low as is usually reported for dry-cured ham [22,30] but some of the samples showed high values of this parameter.

The flavor characteristics included 13 odor, flavor and taste parameters, i.e., a higher number than that usually reported for dry-cured meat [2,7,29,32]. Odor and flavor intensity, cured odor, and flavor and after taste showed mean values close to those reported by Rodriguez-Lazaro et al. [2] and higher than those reported by Molinero Sastre et al. [29]. The mean value for rancid odor was similar to that reported by Lorenzo and Carballo [32], and the rancidity that was perceived during consumption was slightly higher than the rancid odor. Moldy odor has been used for describing other dry meat products [33] and has been pointed out as one of the typical odor notes of this product owing to the presence of *Penicillium* and *Aspergillus* on the surface of the product [34]. Cecina is characterized by its smoked flavor and slightly salty taste [35] which justify the values close to 5 observed for these two parameters (4.90 and 4.35, respectively). These values were slightly higher than those previously found for dry-cured beef cecina [2]. Sweetness or a sweet taste, which is related to the amount of amino acid released during the maturation process [36], is a frequent descriptor of dry-cured ham [7,30] which gives in general higher values than those observed for cecina. Finally, the pungent or burnt taste related to the presence of aldehydes and ketones released from lipid oxidation [36] showed low values.

The texture parameters of hardness, juiciness and chewiness showed a range of variation within the margins reported by Rodríguez-Lázaro et al. and Molinero Sastre et al. [2,29], while fibrousness was lower and juiciness was higher than the mean values reported by these authors and within the margin found by Lorenzo and Carballo [32]. The fatness sensation which has been previously reported as an important descriptor of dry-cured meat products [33] showed a higher mean value and a wider range of variation than juiciness.

### 3.2. Discrimination of the Samples According to Protected Geographical Indication (PGI) Quality Label

“Cecina de León” has the PGI quality label; only the products manufactured in León (Castilla y León, Spain) which follow the scheme approved by the PGI Supervisory Council can bear the PGI label. However, cecina is also produced outside of the PGI but in the same province (León) and following similar manufacturing techniques; therefore, it is necessary to have tools that can help to distinguish whether a product belongs to the PGI or not.

**Figure 2:**
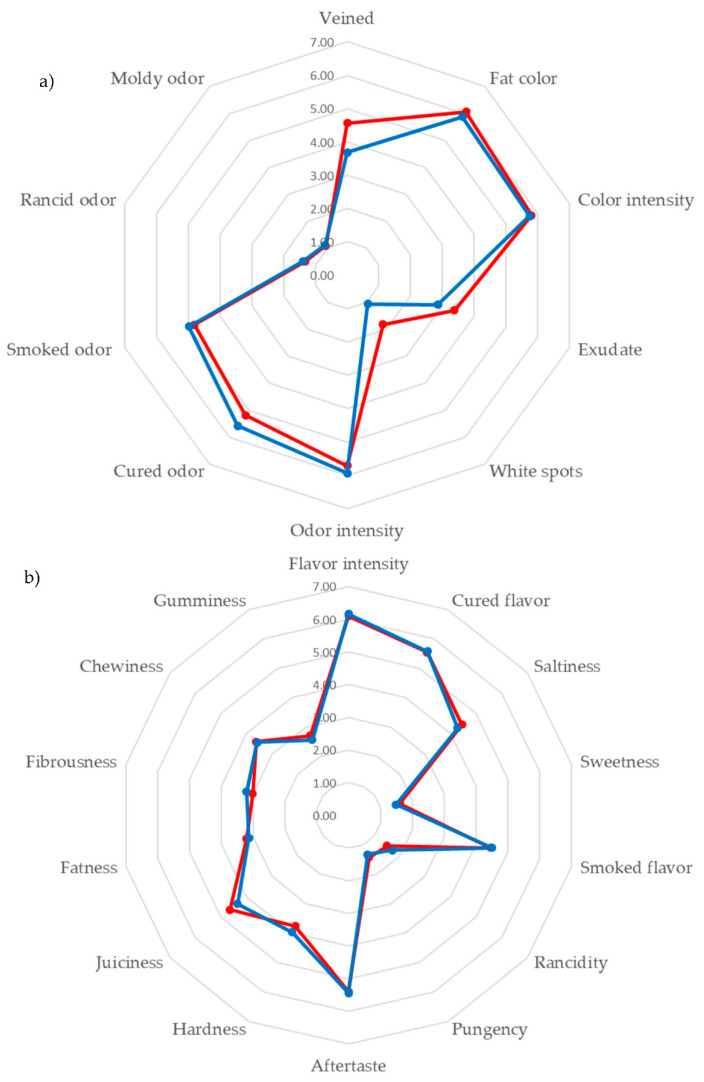
The mean values of (**a**) the appearance and odor attributes and (**b**) flavor and texture attributes of the Protected Geographical Indication (PGI) (red) and non-PGI (blue) samples.

As far as we know, no studies exist comparing the sensory characteristics of cecina depending on whether the product bears the PGI label. The sensory profile of both groups of cecina, PGI cecina de León and non-PGI, is shown in Figure 2.

The statistical analysis of the sensory parameters showed that there were significant differences between PGI and non-PGI cecina in only two parameters: white spots (*p* = 0.019), with the PGI cecina de León samples giving a slightly higher value than non-PGI cecina (1.83 vs. 1.06), and cured odor (*p* = 0.044). In this case, however, the samples from the cecina de León PGI showed a lower value (5.18 vs. 5.58). Veined tended (*p* = 0.067) to be higher in cecina de León PGI samples (4.57 vs. 3.68) but the differences were statistically significant at 90% level. These results show that differences between the sensory characteristics of both groups are small. This is due to the fact that both “cecina de León” and not-PGI cecina are produced from four different pieces of hind leg: thick flank, rump, silver side and topside, that show significant differences in sensory properties [2] and physico-chemical composition [29]. Moreover, the production area is very small and sometimes the same producer manufactures cecina with and without the PGI label.

Therefore, it is very important to have fast and reliable tools that allow the classification of the samples according to their origin. Previous studies have shown that a combination of NIR spectroscopy and chemometric tools is very useful for this purpose [9,10,11,16].

**Figure 3:**
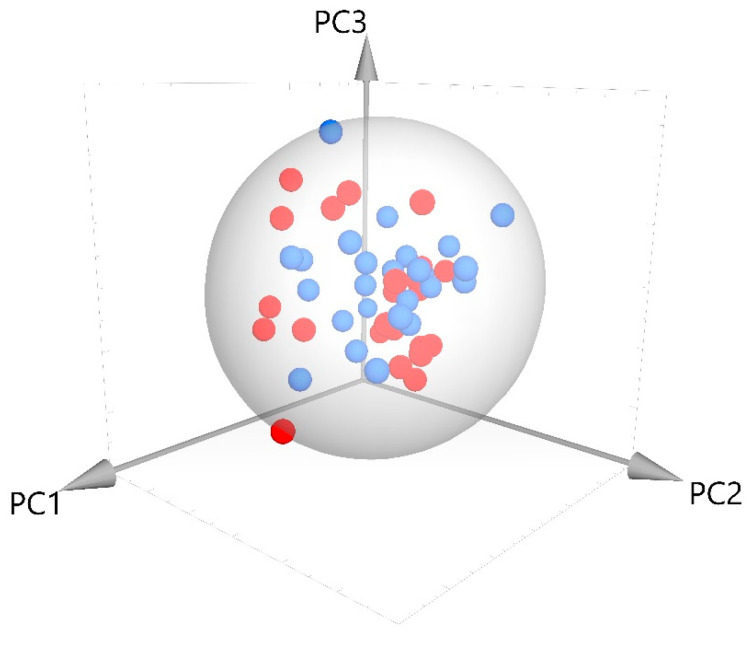
Projection plot of the samples in the space defined by the firsts three components.

The analysis of the whole NIR spectra using SIMCA shows that after the Principal Component Analysis 4 PCs with an eigenvalue > 1 were obtained which explained the 98.8% of the total variance. The projection plot of the samples on the space defined by the three first PCs, which accounts for the 95.5% of the variance shown in Figure 3. It can be observed that both groups are not well separated. In fact, the classification obtained by OPSL-DA (orthogonal projection latent structure discriminant analysis) showed that the 68.4% of the PGI samples and the 70% of non-IGP samples were correctly classified in the calibration and only a 25% of the IGP samples and the 40% of the not-IGP samples were correctly classified in the external validation.

An analysis of the whole NIR spectra using the RMS-X residuals was also carried out. This analysis implies the pre-treatment of the spectra with different combinations of mathematical treatments (MSC, SNV, DT or SNV-DT), derivatives, and smoothing procedures. The optimal treatment is that giving the highest percentage of correctly classified samples. In this case two treatments, None 2,10,10,1 and detrend 2,10,10,1 (Table 2), classify correctly 100% of the samples of non-PGI and 84% of the samples of the cecina de León PGI. The average success rate of the procedure is 92%, which indicates that it is a promising technique as the samples analyzed are very similar to each other.

**Figure 4:**
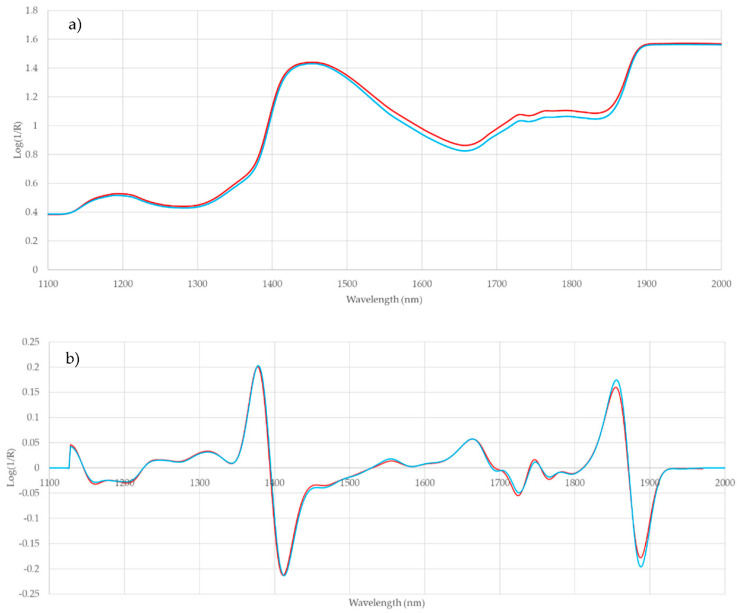
Plot of average near infrared (NIR) spectra of Protected Geographical Indication (PGI) (red) and non-PGI (blue) samples (**a**) without processing and (**b**) processed with detrend 2,10,10,1 mathematical treatment.

The differences in the average spectra of PGI and non PGI samples in the absorption bands are shown in Figure 4a. These differences are due to physical and chemical changes that occur during the maturation process of the cecina. The different treatments that have been applied to correct the scattering of the spectra (None, SNV, DT, SNV-DT) and to achieve the optimization of the discrimination among samples using the RMS-X residual method give rise to different results, as can be seen in Table 2. The differences are due to the fact that the scattering treatments are conditioned by the moisture content of the samples, both of the two groups PGI and non-PGI, moisture that influences the size of the particles and the variations in homogeneity.

Figure 4b plots the average NIR spectra of PGI and non-PGI samples processed with detrend 2,10,10,1 mathematical pre-treatment in which differences between the two groups are observed at certain wavelengths.

Thus, the bands at 1450 nm are correlated with the first overtone of the O-H bond of the water, i.e., with the moisture of the samples. Previous works show that in meat products the 1450 nm band has been mainly related to moisture content; as well as to the third overtone of the C=O bond (1450 nm); and to the first overtone of the NH bond (urea at 1460 nm and CONH_2_ at 1463 [37,38].

On the other hand, a correlation between NIR spectra and C–H–oil and C–O–oil groups at the wavelengths 1720 and 1760 nm, in agreement with the differences observed between PGI and non-PGI samples for veined. Furthermore, the odor intensity which also showed significant differences between the two groups of samples, and which depend on the amount of volatiles such as ketones, aldehydes and other compounds that are produced from lipolysis and fat oxidation [36] are related to the C=O bonds of the ketones at 1896 nm and to the C–H bond of aromatic structures at 1686, 1690 and 1696 nm. Finally, the salt content is reflected in the C–Cl bond at 1856 nm.

Applying ANNs for discriminating the samples, it was possible to find several ANNs with a classifying capability of over 90% for all the learning algorithms assayed. However, it is noteworthy that the number of successful ANNs was low for all of them. The results for the best ANN architecture for the nine learning algorithms, the number of the neurons in the hidden layer and the percentage of the samples correctly classified for the training, validation and test set, together with the average percentage for the total of the samples are shown in the Table 3.

The results show that the gradient descent, gradient descent with adaptive learning rate, gradient descent with momentum, gradient descent with momentum and adaptive learning rate algorithms showed the lowest values of the samples correctly classified ranging from 90.6% to 96.9%. The group of the learning algorithms which uses variations of the conjugate gradient (scaled conjugate gradient, conjugate gradient with Powell–Beale restarts, conjugate gradient with Fletcher–Reeves restarts and conjugate gradient with Polak–Ribiere restarts) obtained higher values of correctly classified samples of between 96.9% and 98.9%. However, Levenberg-Marquardt was the method giving the best results because it was possible to find a significantly higher number of ANN architectures which correctly classified over 90% of the samples and it was also possible to find ANN architectures which correctly classified 100% of the samples.

These results showed the feasibility of the fast and accurate classification of unknown samples according to their origin using NIR spectroscopy.

### 3.3. Prediction of the Sensory Parameters of Cecina

The best ANN architecture, which is obtained by the higher RSQ and the lower MSEP, used for the prediction of cecina the sensory parameters is shown in Table 4. As previously reported, the Levenberg–Marquardt algorithm using the hyperbolic tangent sigmoid for the hidden layer and the linear functions for the output layer is the most suitable for this purpose. With this algorithm, it is possible to find an ANN with a higher RSQ and a lower MSEP than with other algorithms such as gradient scalar. The network was optimized as follows: the data were divided into a training set with 34 samples which was used to obtain the predicting neural network. The accuracy of the ANN (a comparison of the reference with the predicted value) is given by the RSQ. The validation set, which included 8 new samples, was subsequently used to avoid the overfitting of the network. The test set constituted by 8 new different samples was used to check the performance of the network so that new RSQ and MSEP values were obtained. These parameters were used to select the most suitable ANN., so this process was repeated using from 1 to 30 neurons in the hidden layer and for each architecture up to 100 training sessions with different and known seed values were carried out because previous works [23] have shown that the higher the number of trainings the better the prediction capacity of the ANN. The number of neurons for the best ANN architecture, together with the RSQ and the MSEP for the prediction of all the sensory parameters analyzed and for the total of the samples analyzed, is shown in Table 4.

The results show that it was possible to predict all the sensory parameters with very high RSQ values (>0.80) with the exception of odor intensity. These values were higher than those previously reported for the prediction of sensory parameters in meat [39,40,41] and meat products [16,23]. The highest RSQ were observed for white dots (0.99) while odor intensity showed the lowest (0.68), which coincides with that reported by Hernández-Jiménez et al. [16] for dry sausages. In general, the highest RSQ values were observed in texture parameters as previously observed in cheeses by Curto et al. [42], with values close to or higher than 0.9, followed by the appearance parameters with RSQ values close to or higher than 0.85.

**Figure 5:**
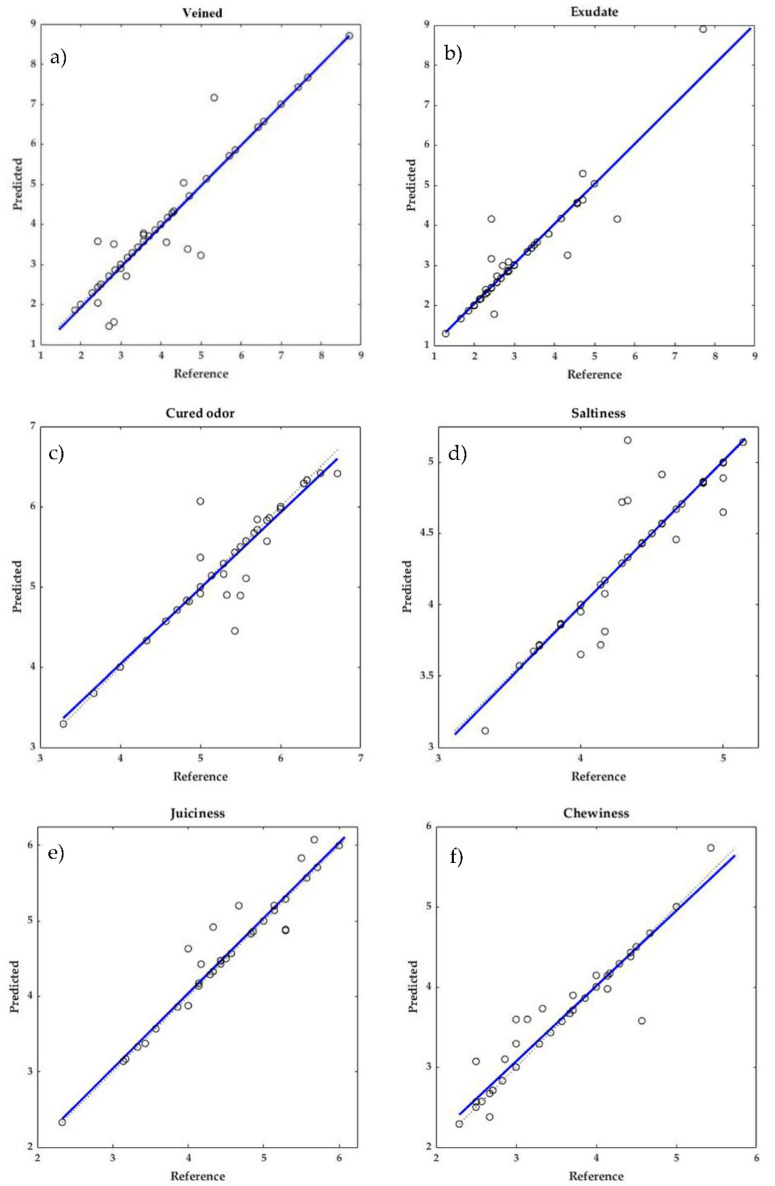
Reference vs. predicted values for (**a**) veined, (**b**) exudate, (**c**) cured odor, (**d**) saltiness, (**e**) juiciness, and (**f**) chewiness.

The MSEPs varied between 0.005 for moldy odor and 0.293 for veined and showed lower values than those previously found for dry-cured ham [23]. Furthermore, as in this study the lowest values of MSEP were observed in general for flavor parameters while the highest values were those of appearance attributes. The small differences between the reference and the predicted values can be seen in Figure 5 in which both sets of data are compared for some of the sensory parameters predicted.

These results point out the t feasibility of the prediction of sensory parameters of dry-cured beef meat cecina using NIR spectroscopy, as a fast and non destructive method, together with ANN using the Levenberg–Marquardt algorithm after the correct optimization process of the ANN.

## 4. Conclusions

From the results obtained it can be concluded that near infrared spectroscopy together with artificial neural networks allow the accurate prediction of almost all (23 out of 24) the sensory parameters selected for an exhaustive characterization of dry-cured beef meat cecina quality with RSQ values higher than 0.8. Taking into account that a sensory analysis is compulsory for cecina de León PGI products, this result stresses the fact that it would possible to substitute the sensory panel by a faster, reliable, non destructive and cheaper instrumental technique that may be implemented on site. Moreover, after an optimization procedure, the ANNs also allow differentiation between the PGI and non-PGI samples produced in the same geographical area with 100% of samples being correctly classified, while the average percentage of correct classification when RMS-X residual was applied was 92%. This reveals that NIRS technology can be a powerful tool to ensure the quality of the product and to prevent fraud.

## Figures and Tables

**Table 1:** Mean, minimum, maximum and standard deviation for 50 samples of cecina.

	Mean	Minimum	Maximum	SD
Appearance				
Veined	4.11	1.86	8.71	1.68
Fat color	5.97	4.43	7.50	0.59
Color intensity	5.78	3.57	8.00	1.09
Exudate	3.11	1.29	7.71	1.15
White spots	1.43	1.00	7.57	1.14
Flavor				
Odor intensity	5.83	4.14	7.00	0.56
Cured odor	5.39	3.67	6.71	0.67
Smoked odor	4.90	3.14	7.00	0.72
Rancid odor	1.36	1.00	3.00	0.40
Moldy odor	1.11	1.00	2.33	0.23
Flavor intensity	6.14	4.00	7.14	0.62
Cured flavor	5.56	3.67	6.86	0.74
Saltiness	4.35	3.33	5.14	0.42
Sweetness	1.54	1.00	2.29	0.30
Smoked flavor	4.49	2.33	5.86	0.71
Rancidity	1.61	1.00	3.83	0.57
Pungency	1.38	1.00	2.00	0.25
Aftertaste	5.42	3.29	6.71	0.58
Texture				
Hardness	3.88	2.33	6.43	0.94
Juiciness	4.49	2.33	6.00	0.81
Fatness	3.17	1.50	6.29	0.96
Fibrousness	3.11	1.71	5.29	0.78
Chewiness	3.60	2.29	5.43	0.84
Gumminess	2.63	1.67	4.50	0.70

**Table 2:** Discrimination results (number of samples and percentage of samples correctly classified) of residual mean squares (RMS-X) residuals method for some of the mathematical treatments assayed.

	None 2,4,4,1		None 2,10,10,1		SNV 1,4,4,1		Detrend 1,4,4,1		Detrend 2,10,10,1	
	PGI	Not PGI	PGI	Not PGI	PGI	Not PGI	PGI	Non-PGI	PGI	Not PGI
PGI	18	7	21	4	22	3	18	7	21	4
Non-PGI	0	25	0	25	3	22	2	23	0	25
Hit rate	72%	100%	84%	100%	88%	88%	72%	92%	84%	100%

PGI: Samples from Protected Geographical Indication, Non-PGI: Samples not belonging to Protected Geographical Indication, SNV: Standard Normal Variate.

**Table 3:** Architecture and discrimination results of the best artificial neural network (ANN) find for each of the assayed learning methods.

	Neurons	Percentage of Samples Correctly Classified			
		Training Set	Validation Set	Test Set	Total
Gradient Descent	27	95.6	85.7	85.7	92.7
Gradient Descent with Adaptive Learning Rate	30	98.5	85.7	85.7	94.8
Gradient Descent with Momentum	9	89.7	100	85.7	90.6
Gradient Descent with Momentum and Adaptive Learning Rate	19	98.5	85.7	100	96.9
Scaled Conjugate Gradient	29	98.5	100	100	98.9
Conjugate Gradient with Powell-Beale	10	100	100	92.8	98.9
Conjugate Gradient with Fletcher-Reeves	18	98.5	100	85.7	96.9
Conjugate Gradient with Polak-Ribiere	7	98.5	100	100	98.9
Levenberg-Marquardt	13	100	100	100	100

**Table 4:** The number of neurons in the hidden layer, correlation coefficient R squared (RSQ), and mean square error or prediction (MSEP) of the best ANN for each sensory parameter.

	Neurons	RSQ	MSEP
Appearance			
Veined	15	0.90	0.293
Fat color	18	0.84	0.054
Color intensity	8	0.89	0.135
Exudate	13	0.87	0.190
White dots	1	0.99	0.008
Flavor			
Odor intensity	9	0.65	0.133
Cured odor	14	0.87	0.066
Smoked odor	25	0.73	0.183
Rancid odor	9	0.84	0.025
Moldy odor	6	0.91	0.005
Flavor intensity	22	0.80	0.097
Cured flavor	14	0.81	0.108
Saltiness	7	0.83	0.037
Sweetness	6	0.83	0.014
Smoked flavor	8	0.81	0.101
Rancidity	25	0.87	0.044
Pungency	25	0.79	0.013
Aftertaste	12	0.88	0.042
Texture			
Hardness	13	0.90	0.090
Juiciness	24	0.95	0.036
Fatness	19	0.90	0.101
Fibrousness	9	0.88	0.067
Chewiness	18	0.92	0.050
Gumminess	15	0.93	0.033
